# An Evaluation of Socio-Demographic and Risk Factor Profile in End-Stage Renal Disease Patients: A Cross-Sectional Assessment

**DOI:** 10.7759/cureus.16353

**Published:** 2021-07-13

**Authors:** Pramod Singh, Abdul Rafae Faisal, Ateeq U Sheikh, Mohammad M Alam, Muhammad Faizan, Purushottam Neupane, Muhammad Uzair, Ayushma Acharya, Ahmad Saeed, Faisal N Akhtar

**Affiliations:** 1 Nephrology Department, Faisalabad Medical University, Faisalabad, PAK; 2 Nephrology Department, Akhtar Saeed Medical and Dental College, Lahore, PAK; 3 Nephrology Department, Allama Iqbal Medical College, Lahore, PAK; 4 Emergency Medicine, Helping Hands Community Hospital, Kathmandu, NPL; 5 Internal Medicine, PNS Hafeez Naval Hospital, Islamabad, PAK

**Keywords:** end stage renal disease (esrd), cross-sectional, diabetes mellitus, hypertension, risk factors

## Abstract

Background

The global incidence and prevalence of chronic kidney disease (CKD) is skyrocketing. In Asia, the prevalence of CKD varies from 10%-18%. However, as Asia is largely populated by developing countries with nascent health care systems, there is a dearth of research and data. It is estimated that a large number of cases go unreported. As a result, the exact disease burden remains unclear. The knowledge about risk factors and their proportionate role in CKD is indispensable in regards to patient management and care.

Objective

The early recognition of the most important risk factors for end-stage renal disease (ESRD) is key to early diagnosis, successful treatment, and general heightened awareness regarding CKD. In developing countries, the provision of medical services, in general, and nephrological services, in particular, is wholly inadequate. The insufficiency of solid and regularly updated data compounds the problem. This research study aims to partake in catering to that need.

Methodology

A structured questionnaire was used to obtain quantitative and categorical data from 119 ESRD patients in the nephrology ward, Allied Hospital, Faisalabad through non-probability sampling. Socio-demographic profile of the patients and information regarding the presence or absence of risk factors were collected. The resulting dataset was analyzed using R version 3.6.3 (R Foundation for Statistical Computing, Vienna, Austria) for data visualization and descriptive analysis.

Results

The most common age group for ESRD presentation was 46-60 years (52.1%). Among the 119 ESRD patients, the most frequent risk factor was hypertension with 85.7% of the patients presenting with the condition, followed by diabetes mellitus (DM) in 54.6%, renal calculi in 28.6%, glomerulonephritis (GN) in 31.1%, Family history of CKD in 24.4%, and polycystic kidney disease (PKD) in 5% of the patients. Gender-wise distribution of the patients shows that the proportion of patients with hypertension, renal calculi, and family history of CKD varied very little among the two groups.

Conclusion

In conclusion, our study has reinforced the existing body of knowledge and brought some fresh evidence regarding the prevalence of risk factors in ESRD to light. Hypertension and DM, together, represent the vast majority of cases with ESRD. However, hypertension far outpaces DM as the leading risk factor. Nephrolithiasis was also present in a considerable minority, with a figure much higher than previously reported. Finally, a relatively younger age group (45-60 years) formed the majority of the ESRD patients which is a concerning development. It points to early progression of CKD to ESRD. Long-term adequate control of these risk factors limits disease progression.

## Introduction

The global incidence and prevalence of chronic kidney disease (CKD) is skyrocketing. Kidney diseases have become the ninth leading cause of death in the United States, imposing a financial strain of 47.5 billion dollars in 2010 alone [1,2]. In Asia, the prevalence of CKD varies from 10%-18% [3], which is quite similar to the figures seen in other regions of the world. However, as Asia is largely populated by developing countries with nascent health care systems, there is a dearth of research and data. It is estimated that a large number of cases go unreported. As a result, the exact disease burden remains unclear [3].

CKD is a general term that refers to a variety of medical problems impacting the morphological structure and physiological function of the kidney [4]. It is a gradual process spread out over multiple stages and develops over a number of years. The stage of kidney failure depends upon the glomerular filtration rate (GFR) of that particular patient. Stage 1: Kidney damage with normal or relatively high GFR (≥90 ml/min/1.73 m2) and chronic albuminuria; Stage 2: Mild reduction in GFR (60-89 ml/min/1.73 m2) with kidney damage; Stage 3: Moderate reduction in GFR (30-59 ml/min/1.73 m2); Stage 4: Severe reduction in GFR (15-29 ml/min/1.73 m2); Stage 5: Established nephropathy (GFR <15 ml/min/1.73 m2), permanent kidney replacement therapy, or end-stage renal disease (ESRD) [5]. 

Stage 5 CKD is referred to as ESRD. It affects over 1500 people per million population (ppm) in countries with high prevalence, like Japan, Taiwan, and the United States [6]. Nearly nine out of 10 patients with ESRD receive hemodialysis, a little more than a quarter receive kidney transplantation, and one in 10 receive peritoneal dialysis [7]. In a local study, the prevalence of ESRD was found to be 17.35%. This figure is very concerning as such patients become a tremendous medical, economic, and social problem for themselves, their families, and the society at large. This is especially true for developing countries such as India and Pakistan. 

The common risk factors leading to ESRD in Pakistan include glomerulonephritis (GN) (37%), hypertension (12%), diabetes mellitus (DM (10%), and renal calculi (5%) [8]. Other risk factors including age, race, obesity, proteinuria, hemoglobin level, nocturia, uric acid level, smoking, recreational drug use, and exposure to nephrotoxins also contribute to the disease [9,10]. 

In Pakistan, insufficient attention has been paid to the prevention of kidney diseases. There is a lack of community-based nephrology services. The lack of healthcare infrastructure, limited education, and financial constraints have hamstrung efforts to address these problems [11]. The early identification of risk factors holds immeasurable importance as timely intervention not only reduces the degree of renal damage but also arrests the vicious circle of persistent proteinuria that eventually results in an irreversible loss of renal function. The adverse outcomes include nephropathy, acceleration of coronary vascular diseases, and premature death which have a great societal and economic impact in relatively poorer countries [12].

## Materials and methods

A structured questionnaire containing close-ended questions was used to obtain quantitative and categorical data from 119 ESRD patients in the nephrology ward, Allied Hospital, Faisalabad through non-probability sampling. Socio-demographic profile of the patients and information regarding the presence or absence of risk factors such as DM, hypertension, polycystic kidney disease (PKD), GN, and family history of renal disease were collected. The resulting dataset was analyzed using R version 3.6.3 (R Foundation for Statistical Computing, Vienna, Austria) for data visualization and descriptive analysis.

## Results

Out of 119 patients, 67 (56.3%) were male and 52 (43.7%) were female. The male to female ratio was 1.28:1. Age distribution of patients included four categories (Table 1). The most common age group for ESRD presentation was 46-60 years (52.1%). There were 18 (17.8%) patients each in the below 30 and above 60 age group with the remaining 21 (17.6%) patients belonging to the 31-45 age group.

**Table 1:** Socio-Demographic Distribution of End-Stage Renal Disease Patients

Socio-Demographic Profile	Frequency	Percentage
Age Group		
30 or below	18	15.1%
31-45	21	17.6%
46-60	62	52.1%
61 or above	18	15.1%
Education		
Bachelor's or above	6	5.0%
Intermediate	9	7.6%
Matric or below	38	31.9%
Primary or below	66	55.5%
Gender		
Female	52	43.7%
Male	67	56.3%
Residence		
Rural	53	44.5%
Urban	66	66.0%

Among the 119 ESRD patients, the most frequent risk factor was hypertension with 85.7% of the patients presenting with the condition, followed by DM in 54.6%, renal calculi in 28.6%, GN in 31.1%, family history of CKD in 24.4%, and PKD in 5% of the patients (Table 2). Gender-wise distribution of ESRD patients shows that the proportion of patients with hypertension, renal calculi, and family history of CKD varied very little among the two groups (Table 3). 61.5% of Female ESRD patients were diabetic, while only 49.3% of males were diabetic. Similarly, 38.8% of males had GN, while only 21.2% of females had the condition. PKD as a risk factor for ESRD was present in 3.8% of females and 6% males.

**Table 2:** Common Risk Factors Leading to End-Stage Renal Disease

Risk Factor	Frequency	Percentage
Diabetes		
No	54	45.4%
Yes	65	54.6%
Hypertension		
No	17	14.3%
Yes	102	85.7%
Glomerulonephritis		
No	82	68.9%
Yes	37	31.1%
Renal Calculi		
No	85	71.4%
Yes	34	28.6%
Polycystic Kidney Disease		
No	113	95.0%
Yes	6	5.0%
Family History of Chronic Kidney Disease		
No	90	75.6%
Yes	29	24.4%

**Table 3:** Gender-Wise Distribution of End-Stage Renal Disease Patients

Risk Factor		Female		Male	
Diabetes	No	20	38.5%	34	50.7%
	Yes	32	61.5%	33	49.3%
Hypertension	No	7	13.5%	10	14.9%
	Yes	45	86.5%	57	85.1%
Glomerulonephritis	No	41	78.8%	41	61.2%
	Yes	11	21.2%	26	38.8%
Renal Calculi	No	37	71.2%	48	71.6%
	Yes	15	28.8%	19	28.4%
Polycystic Kidney Disease	No	50	96.2%	63	94.0%
	Yes	2	3.8%	4	6.0%
Family History of Chronic Kidney Disease	No	39	75.0%	51	76.1%
	Yes	13	25.0%	16	23.9%

## Discussion

CKD is an important chronic disease globally [13]. It affects diverse populations all across the world and has become a growing health issue. Hypertension occurs in 72 million people worldwide [14]. In our study, hypertension was found to be the leading risk factor present among ESRD patients. This is also corroborated by other studies [15]. The reported prevalence of hypertension, while higher than in normal individuals for all stages of CKD, reaches astronomical proportions of 84.1% in patients with late stages of CKD (Stages 4 & 5) [16]. This mirrors the results obtained in our study. However, it should be noted that a considerable proportion of these hypertensives will have developed the disease as a consequence of CKD rather than the other way around [17]. Uncontrolled hypertension causes long-term damage to the blood vessels that supply the kidney. This deprives the kidneys of the blood and oxygen supply leading to a reduction in function and, later, failure [18]. 

Along with hypertension, DM is found to be one of the two primary risk factors for ESRD. High serum glucose for a prolonged period can result in damage and clogging of renal blood vessels impacting kidney function [19]. Over half of the patients had DM. This is concurrent with regional statistics from countries such as Taiwan (43.2%) and Hong Kong (46.2%) [20]. GN is the third leading cause of ESRD. It damages the glomeruli, which are the filtering units of the kidney [21]. 

Five percent of ESRD patients in our study had PKD. The results vary when compared to an American study which finds it to be present in 1.5% of the ESRD patients [22]. Renal calculi damage kidney tissue by causing ureteral obstruction, hydronephrosis, and repeated infections. In severe and prolonged cases, it can even lead to kidney failure [23]. In the past, nephrolithiasis was considered inconsequential as a causal factor for ESRD. In fact, the difficulty of establishing causality was the main reason behind this attitude. However, recently, medical opinion has started to shift [24]. Our study has found it to be associated with a significant number of cases (28.6%). While this figure is bigger than previously reported, it also comes with a caveat. More often than not, nephrolithiasis is a contributing factor in CKD along with more important and common causes such as hypertension and DM [25]. Hence, it is more advisable to view the situation as a result of multiple contributing factors that lead to, eventually, a complete loss of kidney function. In our study, there were only five out of 34 ESRD patients with only nephrolithiasis as a risk factor. 

This study puts the proportion at 56.3% for men and 43.7% for women. In another study, out of 1530 patients, 56.14% were men and 43.86% were women [26]. Higher levels of education are associated with better health outcomes. It is no different in the case of kidney diseases. In keeping with that, our study also supports the inverse association of ESRD incidence with education and health [27].

## Conclusions

In conclusion, our study has reinforced the existing body of knowledge and brought some fresh evidence regarding the prevalence of risk factors in ESRD to light. Hypertension and DM, together, represent the vast majority of cases with ESRD. However, hypertension far outpaces DM as the leading risk factor present. Nephrolithiasis was also present in a considerable minority, with a figure much higher than previously reported. Finally, a relatively younger age group (45-60 years) formed the majority of the ESRD patients which is a concerning development. It points to early progression of CKD to ESRD. Long-term adequate control of these risk factors limits disease progression. In addition, a higher level of education and awareness is associated with better health outcomes because such patients are more likely to seek medical attention and receive timely treatment, leading to greater control of the associated risk factors.

## References

[1] Kearns B, Gallagher H, de Lusignan S (2013). Predicting the prevalence of chronic kidney disease in the English population: a cross-sectional study. BMC Nephrol.

[2] Coresh J, Selvin E, Stevens LA (2007). Prevalence of chronic kidney disease in the United States. JAMA.

[3] Hamer RA, El Nahas AM (2006). The burden of chronic kidney disease. BMJ.

[4] Levey AS, Eckardt KU, Dorman NM (2020). Nomenclature for kidney function and disease: report of a Kidney Disease: Improving Global Outcomes (KDIGO) Consensus Conference. Kidney Int.

[5] Levey AS, Stevens LA, Coresh J (2009). Conceptual model of CKD: applications and implications. Am J Kidney Dis.

[6] Collins AJ, Foley RN, Chavers B (2012). US Renal Data System 2011 Annual Data Report. Am J Kidney Dis.

[7] (2021). Kidney disease statistics for the United States. https://www.niddk.nih.gov/health-information/health-statistics/kidney-disease.

[8] Sakhuja V, Sud K (2003). End-stage renal disease in India and Pakistan: burden of disease and management issues. Kidney Int Suppl.

[9] Levin A, Hemmelgarn B, Culleton B (2008). Guidelines for the management of chronic kidney disease. CMAJ.

[10] Tonelli M, Wiebe N, Culleton B (2006). Chronic kidney disease and mortality risk: a systematic review. J Am Soc Nephrol.

[11] Garcia-Garcia G, Jha V (2014). Chronic kidney disease in disadvantaged populations. Nephron Clin Pract.

[12] Levey AS, Coresh J (2012). Chronic kidney disease. Lancet.

[13] Ruggenenti P, Schieppati A, Remuzzi G (2001). Progression, remission, regression of chronic renal diseases. Lancet.

[14] Varon J (2008). Treatment of acute severe hypertension: current and newer agents. Drugs.

[15] London GM, Guerin AP, Pannier B, Marchais SJ, Safar ME (1998). Large artery structure and function in hypertension and end-stage renal disease. J Hypertens.

[16] Tedla FM, Brar A, Browne R, Brown C (2011). Hypertension in chronic kidney disease: navigating the evidence. Int J Hypertens.

[17] Pugh D, Gallacher PJ, Dhaun N (2019). Management of hypertension in chronic kidney disease. Drugs.

[18] Bidani AK, Griffin KA (2004). Pathophysiology of hypertensive renal damage: implications for therapy. Hypertension.

[19] Lopez-Vargas PA, Tong A, Phoon RK, Chadban SJ, Shen Y, Craig JC (2014). Knowledge deficit of patients with stage 1-4 CKD: a focus group study. Nephrology.

[20] Duan J, Wang C, Liu D (2019). Prevalence and risk factors of chronic kidney disease and diabetic kidney disease in Chinese rural residents: a cross-sectional survey. Sci Rep.

[21] Edvardsson VO, Goldfarb DS, Lieske JC, Beara-Lasic L, Anglani F, Milliner DS, Palsson R (2013). Hereditary causes of kidney stones and chronic kidney disease. Pediatr Nephrol.

[22] Abbott KC, Agodoa LY (2002). Polycystic kidney disease at end-stage renal disease in the United States: patient characteristics and survival. Clin Nephrol.

[23] Keddis MT, Rule AD (2013). Nephrolithiasis and loss of kidney function. Curr Opin Nephrol Hypertens.

[24] Rule AD, Krambeck AE, Lieske JC (2011). Chronic kidney disease in kidney stone formers. Clin J Am Soc Nephrol.

[25] Rule AD, Bergstralh EJ, Melton LJ III, Li X, Weaver AL, Lieske JC (2009). Kidney stones and the risk for chronic kidney disease. Clin J Am Soc Nephrol.

[26] Chang PY, Chien LN, Lin YF, Wu MS, Chiu WT, Chiou HY (2016). Risk factors of gender for renal progression in patients with early chronic kidney disease. Medicine.

[27] Morton RL, Schlackow I, Staplin N (2016). Impact of educational attainment on health outcomes in moderate to severe CKD. Am J Kidney Dis.

